# Evaluation of continuing education of family health strategy teams for the early identification of suspected cases of cancer in children

**DOI:** 10.1186/s12909-017-0993-1

**Published:** 2017-09-07

**Authors:** Ana Maria Aranha Magalhaes Costa, Cynthia Magluta, Saint Clair Gomes Junior

**Affiliations:** Unidade de Pesquisa Clínica, Instituto Nacional de Saúde da Mulher, da Criança e do Adolescente Fernandes Figueira, Fundação Oswaldo Cruz ( Fiocruz ), Av. Rui Barbosa 716 Flamengo, Rio de Janeiro, 22250-020 Rio de Janeiro Brazil

**Keywords:** Continuing education, Early detection of cancer, Child, Non-governmental organization (NGO), Brazil, Childhood cancer

## Abstract

**Background:**

This study evaluated the influence of continuing education of family health strategy teams by the Ronald McDonald Institute program on the early diagnosis of cancer in children and adolescents.

**Methods:**

The study applied Habicht’s model to evaluate the adequacy and plausibility of continuing education by using as outcome the number of children with suspected cancer who were referred to the hospital of references in the 1 year before and 1 year after intervention and the number of patients referred by intervention group and control group family health strategy teams. Medical records from each hospital of reference were used to collect information of suspect cases of cancer. Descriptive analyses were performed using frequencies and mean values. Chi-square tests were used to assess statistically significant differences between the groups and periods by using *p*-values < 0.05.

**Results:**

The results showed a 30.6% increase in the number of children referred to the hospital of reference for suspected cancer in the post-intervention period; in addition, the family health strategy teams that underwent the intervention referred 3.6 times more number of children to hospital of references than did the control group. Only the intervention group showed an increase in the number of confirmed cases.

**Conclusions:**

This evaluation of a continuing education program for early identification of pediatric cancer showed that the program was adequate in achieving the established goals and that the results could be attributed to the program.

## Background

Cancer in children and adolescents in Brazil accounts for 3% of cases of neoplasia, excluding non-melanoma skin cancer, with an estimated 12,600 new cases in children and adolescents up to 19 years of age in 2016 [1]. Cancer is considered a public health problem, as it is the leading cause of death (8% of the total) due to illness between 1 and 19 years old population, with a total of 2835 deaths in 2013. The most frequent tumors in childhood and adolescence are leukemias, central nervous system and lymphomas. Neuroblastoma, Wilms’ tumor, retinoblastoma, germ cell tumor, osteosarcoma, and sarcomas also is observed in this population [1, 2].

The signs and symptoms of cancer may be nonspecific and can be mistaken for those of more common diseases in this age group, thus delaying the diagnosis and referral of children to hospitals of reference who then arrive with advanced stages of the disease. A lack of information in the society and among health professionals in considering this nosological entity in the differential diagnoses is one of the reasons for its late detection [3].

Progress in cancer treatment in childhood and adolescence has been extremely significant. Today, around 80% of children and adolescents with cancer can be cured, if diagnosed early and treated in specialized centers [2]. Early detection of cancer enables the use of less aggressive therapeutic protocols and minimizes sequelae resulting from the disease or treatment [4]. In developed countries, there is a correlation between an increased chance of survival (up to 75%) and early diagnosis [5–8]. Articulation of the health care network and care infrastructure are important factors in the dynamics of the early diagnosis and treatment of children and adolescents with cancer.

Based on the importance of early diagnosis, this study evaluated the influence of continuing education for family health strategy (FHS) teams on the early diagnosis of cancer in children and adolescents. The FHS teams deliver community-based primary care in the Brazilian health system [9]. The nucleus of each FHS team includes, at least, a physician, a nurse, a nurse assistant, and four to six full-time community health agents, all these professionals are qualified to identify signs and symptoms of childhood illnesses, including cancer. FHS teams are organized geographically, covering populations of up to 1000 households each, with no overlap or gap between catchment areas [9]. The suspected cases of childhood cancer are referred to hospitals of reference of the same geographic area for diagnostic confirmation and treatment.

Our hypothesis or field contribution was that FHS teams submitted to intervention refer more children and adolescents with suspected cancer to hospitals of reference.

## Method

### Case presentation

The Ronald McDonald Institute (RMDI) is a non-governmental organization (NGO). Its mission is “to promote the health and well-being of children and adolescents with cancer”. Thus, it identifies priority demands in the fight against cancer in children and adolescents and develops supporting programs, including the “Early Diagnosis Program” [10]. The goals of this program are to train FHS teams to identify children with suspected cancer and to reduce the time from first the complaint that resulted in a demand for any health service to the arrival to the first medical oncologist appointment at a hospital of reference. Therefore, the program reinforces FHS professionals to suspect cancer in children and adolescents in order to increase the possibility of an early diagnosis; it also contributes to the organization of the oncological care network in territories where the program has been deployed. The Institute is connected to a network of partners nation wide including municipal health care divisions and NGO working for the same cause in several Brazilian regions, which coordinate the program at a local level.

### Intervention

The intervention was a continuing education program for FHS teams focused on the early identification of suspected pediatric cancer and referral to a hospital of reference. The pedagogical proposal was designed by health education professionals, oncologists at the hospitals of reference and managers of the RMDI. The duration of the intervention was 20 h, distributed in eight modules that were presented as lectures and group exercises by multidisciplinary teams coordinated by oncologists at the eight hospitals of reference of the Unified Health System (SUS in Portuguese). These hospitals responded to postings from the RMDI in 2008 and 2010 to participate as local coordinators of the intervention and were responsible for the selection of the FHS teams for the intervention, using as eligible criteria: to be in the same area of the hospital of reference, cities with less than 50,000 inhabitants or areas with human development index (HDI) less than 80 in cities with more than 50,000 inhabitants. The eligible criteria to intervention group were defined by RMDI priorizing teams from smaller or poorer areas, with less access to knowledge recycling. The FHS teams were considered as reinforced (intervention group) when at least one physician or nurse from each team achieved a 75% participation frequency in the continuing education activities. Control group was composed of FHS teams from areas that did not have the eligibility criteria or teams that did not reach 75% of frequency.

The eight modules were: epidemiology and organization of the cancer care network; the importance of the FHS team for early detection and monitoring of children and adolescents with cancer; rights of children and adolescent with cancer; possibilities and limitations of early detection; signs and symptoms of cancer in childhood and adolescence; health care for children and adolescent with cancer; palliative care in pediatric oncology; and psychology care of child and adolescent with cancer.

### Evaluation of the intervention

The intervention was assessed based on an evaluation model for public health programs proposed by Habicht et al. [11]. This model evaluates the effectiveness of large-scale programs and was chosen for this study because the characteristics of the RMDI program actions resemble the actions of collective health programs. The model proposes an evaluation of adequacy and plausibility. The first criterion evaluates the performance of the program by measuring how well it meets pre-established goals, while the second establishes the causal relation between the intervention and the changes that took place [11].

### Outcome of the intervention

In this study, adequacy was assessed by comparing the number of children with suspected cancer who arrived at the hospital of reference in the pre- and post-intervention periods. Plausibility was assessed in this study by comparing the number of children with suspected cancer who arrived at the hospital of reference and were referred by intervention or control groups. In addition, it was compared the performances of intervention and control groups in the pre- and post-intervention periods in terms of the number of suspected cases of cancer referred to the hospital of references and the number of cases with confirmed diagnoses.

### Period of the intervention

The interventions occurred in 2008 and 2010 with different groups of participants. For the intervention group in 2008, the years 2007 and 2009 were considered the pre- and post-intervention periods, respectively. Similarly, 2009 and 2011 were the pre- and post-intervention periods for the intervention group in 2010.The control group was assessed at the same time points as the intervention group. The period of 1 year before and 1 year after intervention was selected in order to minimize seasonality due to the local organization.

### Sample, data collection variable

The sample was composed by all medical records of children referred for the first time to the hospital of reference for suspected cancer. Data were collected in the clinical report of the study from medical records and documents of hospitals of reference. Variables observed were: demographic data (gender, age and address) and confirmation of diagnosis (cancer or non-cancer) were observed. The residential addresses of the children were used to determine if they were referred by intervention or control groups.

### Statistical analysis

Descriptive analyses were performed to obtain a profile of the study population using frequencies and means. Chi-square tests were used to assess statistically significant differences between the groups and periods. The data were registered in Epi-Info version 3.5.3 and analyzed using SPSS Statistics for Windows, version 17.0. All analysis was performed at a significance level of 0.05.

### Ethical issues

All participants in this study signed an informed consent form (ICF) after receiving a detailed explanation of the research. All data were collected with consent to the head of the of hospital of reference, which participated in the study.

The study was approved by the Ethics Committee (EC) of each participating center and by the EC of the coordinating center, under registration number 0071.1.008.000–11; the study was developed at the request of and with financing from the RMDI.

## Results

The sample consisted of 1797 children and adolescents with a mean age of 7.9 years (±5.2); of them, 988 (55%) were male. There were 779 (43.3%) children in the pre-intervention period and 1018 (56.7%) children in the post-intervention period. Among all children and adolescents in the sample, 446 (25%) were from the intervention group. Diagnoses were confirmed in 51.3% of cases (Table 1).

**Table 1:** Study sample characteristics: demographic data, period, group, and diagnosis

		*n* = 1797	Mean ± sd
Age (years)			7.9 ± 5.2
Sex	Male	988 (55.0%)	
	Female	809 (45.0%)	
Period	Pre	779 (43.3%)	
	Post	1018 (56.7%)	
Group	Intervention	446 (25.0%)	
	Control	1351 (75.0%)	
Diagnosis	Confirmed	922 (51.3%)	
	Unconfirmed	875 (48.7%)	

*sd* standard deviation

Analysis of the adequacy of the program according to the number of children and adolescents with suspected cancer that were referred to a hospital of reference revealed a total of 779 and 1018 children referred in pre-and post-intervention periods, which represented an increase of 239 (30.6%) children referred in the post period. However, this increase was not followed by a similar proportion of increase in the number of diagnosed cases. The percentage of confirmed diagnoses in the pre-intervention period was 49.4%, compared to 50.5% in the post-intervention period, an increase of 10 cases (2.2%) (Table 2).

**Table 2:** Adequacy: children with suspected cancer referred to hospitals of reference according period and diagnosis

Period of evaluation	Children with suspected cancer that were referred						*P* value
	Confirmed		Not confirmed		Total		
	n	%	n	%	n	%	
Post	466	50.5	552	63.1	1018	56.7	<0.0001
Pre	456	49.4	323	36.9	779	43.3	
Total	922	100	875	100	1797	100	

Analysis of the plausibility based on the number of children and adolescents with suspected cancer who wer referred to a hospital of reference did not confirm the hypothesis of the study (Table 3).

**Table 3:** Plausibility: Children with suspected cancer referred to hospitals of reference by intervention and control groups

Group	Children with suspected cancer that were referred						*P* value
	Confirmed		Not confirmed		Total		
	n	%	n	%	n	%	
Intervention	87	18.7	195	37.4	282	27.7	<0.0001
Control	379	81.3	357	62.6	736	72.3	
Total	466	100	522	100	1018	100	

Stratification of the FHS teams over the period revealed that the intervention group had an increased number of cases referred for suspected cancer in the post-intervention period (118 cases, 71.9%). This increase was followed by an increase of nine cases (11.1%) with confirmed diagnosis. In comparison, the control group had an increase of 121 cases referred for suspected cancer (19.6%) in post-intervention period and one case (0.2%) of confirmed diagnosis (Table 4). This stratification allowed us to confirm the hypothesis of this study.

**Table 4:** Comparison of intervention and control groups performance pre- and post- intervention

Group	Period of evaluation	Diagnosis of referred children						*P* value^*^
		Confirmed		Not confirmed		Total		
		n	%	n	%	n	%	
Intervention	Post	87	52.7	195	69.4	282	63.2	0.0013
	Pre	78	47.3	86	30.6	164	36.8	
Total		165	100	281	100	446	100	
Control	Post	379	50.1	357	60.1	736	54.5	
	Pre	378	49.9	237	39.9	615	45.5	
Total		757	100	594	100	1351	100	

^*^calculated as the difference in the percentage of children referred by intervention and control groups in the post-intervention period

## Discussion

The post-intervention period showed a higher number of children with suspected cancer referred to hospitals of reference than those referred in the pre-intervention period, which demonstrates the adequacy of the intervention. Plausibility was evaluated based on the number of patients suspected to have cancer, who arrived at a hospital of reference after being referred by intervention or control groups. This study did not observe a positive relationship for the intervention. The plausibility analysis proposed by Habitch et al. [11] was not sensitive enough to infer causality between intervention and outcome.

Confounding factors such as the pairing between intervention and control groups and migration of professionals between teams may have affected the results. The elegible criteria promoted the imbalance between the groups favoring the number of control group and may explain the low sensitivity of the method described by Habitch et al. [11] to identify the plausibility between intervention and outcome. The migration of professionals between teams is practically impossible to control in the Unified Health System and likely had a marginal effect on the control group. The dissemination of knowledge with the migration of professionals between teams is desirable for this type of intervention. In addition, the inclusion of teams that did not obtain 75% of the frequency in control group may also have contributed to the low sensitivity of the method.

Stratified analysis of the intervention and control groups pre- and post-intervention revealed that intervention group referred 3.6 times more number of children to the hospital of reference than did the control groups. There was an increase in the number of confirmed cases only in intervention group. Stratification showed the association between intervention and outcome and confirmed the study hypothesis that intervention group would refer more children and adolescents with suspected cancer to hospital of references.

These results are in accordance with other studies that also evaluated continuing education which have been used for health care professionals and have proven to be effective in the early detection of cancer. In Central America, a reduction in the detection rate of retinoblastoma in later stages was observed after diagnosis teams completed training programs [12, 13]. Toftegaard et al. carried out a pragmatic clinical trial to evaluate continuing education to standardize the knowledge of general practitioners (GP) for the early diagnosis of cancer. They observed that the GP undergoing the intervention thought more about the diagnosis of cancer after the intervention [14, 15]. Continuing education has also been adopted for other health conditions and has shown a positive effect on the health team’s knowledge, attitude and behavior [16].

In addition to the team training strategy, the participation of NGOs in the program may have also contributed to the results of the current study. Likewise, studies by Costa et al., and Ricca et al.evaluated the influence of NGO actions on the health of patients they assisted, reporting that partnership with these entities was an effective action strategy [17, 18]. An NGO’s capacity to connect with communities facilitates and promotes the implementation and dissemination of interventions for better health [19–23] . The results of cooperation between the third and public sectors have shown its efficacy. Actions such as those supported by IRMD’s program empower teams in the public sector to continue the program even if the provider leaves.

This study had several limitations, including the selection criteria and the observation period. Regarding the selection criteria, the decision to evaluate only children and adolescents who arrived at the hospital of references through the outpatient-screening clinic was made because of operational reasons and up on agreement with the center coordinators, who identified these clinics as the main arrival for patients referred for suspected cancer. This decision could have resulted in the loss of children referred for suspected cancer who entered by other sectors. This decision does not likely affect the estimated number of suspected cases of cancer because these cases were relatively few in number compared to those reporting to the outpatient screening clinic.

The period selected for evaluation of the results (one year before and one year after) may have been too short to allow an assessment of the long-term effects of the intervention, since it does not permit an analysis of the trend of the suspected cases identified in both groups, especially for the post-intervention period.

## Conclusion

This evaluation of RMDI’s Early Diagnosis Program showed that the program was able to achieve the established goals and that the results could be attributed to the program.

The continuing education may be an effective strategy to increase the early diagnosis of cancer in children and adolescents in developing countries where reducing morbidity and mortality in this population is challenging.

The agenda of public policies focused on cancer in children and adolescents should emphasize early diagnosis. Cooperation between the third sector and the public health system could be encouraged to offer continuing education since the RMDI’s experience has shown the effectiveness of these approaches.

## Abbreviations

EC: Ethics Committee
FHS: Family Health Strategy
ICF: Informed Consent Form
NGO: Non-Governmental Organisation
RMDI: Ronald McDonald Institute
SUS: Brazilian Unified Health System
